# Leukocyte telomere length and diet in the apparently healthy, middle-aged Asklepios population

**DOI:** 10.1038/s41598-018-24649-9

**Published:** 2018-04-25

**Authors:** Tim De Meyer, Sofie Bekaert, Marc L. De Buyzere, Dirk D. De Bacquer, Michel R. Langlois, Nitin Shivappa, James R. Hébert, Thierry C. Gillebert, Ernst R. Rietzschel, Inge Huybrechts

**Affiliations:** 1 Department of Data Analysis and Mathematical Modelling, Faculty of Bioscience Engineering, Ghent University, Coupure Links 653, B-9000 Ghent, Belgium; 2 BIMETRA – Clinical Research Center Ghent, Ghent University Hospital, De Pintelaan 185, B-9000 Ghent, Belgium; 3 Department of Cardiovascular Diseases, Ghent University Hospital, De Pintelaan 185, B-9000 Ghent, Belgium; 4 Department of Public Health, Ghent University, De Pintelaan 185, B-9000 Ghent, Belgium; 5 Department of Laboratory Medicine, Asklepios Core-Lab, AZ St-Jan AV Hospital, Ruddershove 10, B-8000 Bruges, Belgium; 6 Cancer Prevention and Control Program, University of South Carolina, 915 Greene Street, Suite 241, Columbia, SC 29208 USA; 7 International Agency for Research on Cancer; 150 Cours Albert Thomas, 69372, Lyon, CEDEX 08 France

## Abstract

Telomere length is a prognostic biomarker for aging diseases. As it is unknown whether diet plays a role in these associations, we aimed to assess the impact of diet on telomere length. Moreover, given that telomere length is modulated by oxidative stress and inflammation, an additional goal was to evaluate whether the latter may mediate possible telomere – diet associations. Southern blot measured leukocyte telomere length and food frequency questionnaire data were compared for 2509 apparently healthy men and women (~35 to 55 years) from the Asklepios population. No significant associations were found between telomere length and overall dietary characteristics, such as dietary diversity, quality, equilibrium, and the dietary inflammatory index. Exploratory analysis of individual dietary variables revealed that a higher daily intake of deep fried potato products was associated with shorter telomeres (*P* = 0.002, 151 bp per 100 g/day), also in both sexes separately. Deep fried potato product consumption was also significantly associated with C-reactive protein (*P* = 0.032) and uric acid (*P* = 0.042), but not other inflammation and oxidative stress markers. These results suggest an at most limited association between overall dietary patterns and telomere length in the general population. Nevertheless, the association between telomere length and deep fried potato product intake warrants additional research.

## Introduction

Chromosomal ends are distinguished from double-stranded breaks by a complex nucleoprotein structure, the telomere. Telomeres shorten with each cell division due to the end-replication problem, until critical shortening occurs and replicative senescence is induced. Epidemiologic studies have demonstrated that inflammation, oxidative stress and related lifestyles are associated with lower average telomere length, typically measured in peripheral blood leukocytes (PBL). This has led to the hypothesis that an individual’s leukocyte telomere length (LTL), although primarily inherited, also chronicles the cumulative burden of intrinsic and lifestyle-generated inflammation and oxidative stress (reviewed by De Meyer *et al*.^1^). LTL has been observed as an independent prognostic marker for cardiovascular diseases, cancer and mortality due to infectious disease^2–4^, supporting systemic impact of (critically) short telomeres. Importantly, genetic determinants of LTL are also independently associated with cancer and cardiovascular risk, suggesting causality^5–7^.

The preponderance of the evidence suggests that the effects of inflammation and oxidative stress on successful aging are, in part, mediated by LTL. Although the exact mechanisms that involve LTL in aging diseases remain to be established, several putative lifestyle-related factors influencing telomere attrition have been proposed. These include BMI (body mass index)/obesity, smoking, and physical activity^8–10^. Specific diet/nutrition related factors reported to be associated with LTL include dietary fiber, omega-3 and omega-6 polyunsaturated fatty acids, Mediterranean diet adherence, saturated fatty acid subgroups, processed meat, vitamin D, sugar-sweetened beverage, multivitamin and overall energy intake^11–21^. Diets characterised by a high antioxidant or anti-inflammatory capacity have been associated with longer telomeres^22–24^. On balance, however, little information is available regarding the effects of general dietary patterns on telomere biology, particularly in the general population.

The Asklepios Study on successful (cardiovascular) aging, which includes data on over 2500 men and women, provides an excellent tool to study this. In this population, no associations between LTL and typical lifestyle factors as smoking, BMI, alcohol consumption and physical activity were found, most likely due to the generally healthy nature of the population. On the other hand, oxidative stress and inflammatory markers were significantly associated with LTL^25^. Therefore, other lifestyle related factors might be more important and underlie these associations. As the dietary profile of the Asklepios study population has been described using a semi-quantitative food frequency questionnaire^26^, these data were used here to assess their association with LTL.

This study was designed to investigate associations between dietary indicators available in the Asklepios baseline measurements and LTL, with a particular focus on the overall dietary pattern. In addition, we also aim to assess the associations between specific individual food groups and LTL and to evaluate whether detected diet - LTL associations may be modulated by inflammation and oxidative stress.

## Results

### LTL and overall dietary characteristics

Baseline characteristics of the population under study, including diet and oxidative stress and inflammation related parameters, are summarised in Tables 1 and 2. For each variable, information was available for at least 72% of all subjects. As reported previously^25,27^, in this population LTL is inversely associated with subject’s age, positively with paternal age at conception, and shorter in men than in women (GLM, each variable *P* < 0.0001). Upon adjustment for these confounders, no significant associations could be identified between LTL and holistic dietary patterns, i.e. overall dietary score, dietary quality, dietary diversity and dietary equilibrium (Table 3). Additionally, also the association between LTL and other general dietary characteristics, i.e. total daily energy, fiber intake, as well as the dietary inflammatory index (DII) were evaluated, yet without significant results (Table 3). Note that earlier analyses found no association between LTL and resp. BMI, obesity and weight in this population^25^.

**Table 1:** Baseline population and biochemical characteristics.

Variable	Women (N = 1291)	Men (N = 1218)	Sign. Diff.
***Subject characteristics (see also*** ^25^ ***and*** ^27^ ***)***			
Age (years)	45.9 (6.0)	46.1 (5.9)	*P* = 0.32
Weight (kg)	66.7 (12.7)	82.0 (12.4)	*P* < 0.001
BMI (kg/m²)	25.1 (4.6)	26.5 (3.7)	*P* < 0.001
Obesity (individuals, %)	175 (13.6%)	211 (17.3%)	*P* = 0.009
Telomere length (kbp)	7.96 (0.73)	7.78 (0.71)	*P* < 0.001
Paternal age at birth offspring (years)	31.5 (6.5)	31.8 (6.7)	*P* = 0.39
Physical activity (METs)	0 [0–4.6]	0 [0–14.2]	*P* < 0.001
***Biochemical marker concentrations (see also*** ^25^ ***)***			
OxLDL (U/L)	92 (38)	101 (39)	*P* < 0.001
hs-CRP (mg/L)	1.42 [0.62–3.42]	1.05 [0.56–2.04]	*P* < 0.001
IL-6 (pg/mL)	0.75 [0.00–1.50]	0.79 [0.00–1.60]	*P* = 0.29
Fibrinogen (mg/dL)	336 (65)	314 (59)	*P* < 0.001
Serum uric acid (mg/dL)	4.32 (1.05)	6.06 (1.27)	*P* < 0.001

Data indicate mean (standard deviation), median [interquartile range] or frequency (percentage), and significance of difference as respectively tested by Student t-test (or Welch’s t-test, if Levene’s test for homoscedasticity yielded *P* < 0.05), Wilcoxon rank sum test and Fisher’s Exact Test.

**Table 2:** Baseline dietary characteristics.

Variable	Women (N = 1291)	Men (N = 1218)	Sign. Diff.
***General dietary characteristics (see also*** ^26^ ***)***			
Overall dietary score (%)	69.3 (12.9)	58.8 (14.8)	*P* < 0.001
Diversity score (%)	79.4 (11.3)	76.1 (11.1)	*P* < 0.001
Quality score (%)	54.3 (30.1)	30.6 (32.2)	*P* < 0.001
Equilibrium score (%)	72.8 (9.5)	69.7 (10.3)	*P* < 0.001
Energy intake (MeJ/day)	8.12 (2.23)	9.61 (2.55)	*P* < 0.001
Fiber intake (g/day)	25.3 (7.02)	25.7 (8.11)	*P* = 0.24
DII score	−1.02 (0.77)	−0.92 (0.74)	*P* = 0.001
***Average daily intake food items***			
Sweetened or alcoholic beverages (mL)	337 (352)	651 (441)	*P* < 0.001
Non-sweetened beverages (mL)	950 (460)	841 (486)	*P* < 0.001
Whole milk or sweetened milk products (mL)	68.3 (127.0)	90.3 (139.0)	*P* < 0.001
(Semi-)skimmed unsweetened milk products (mL)	196 (194)	130 (173)	*P* < 0.001
Fruits (g)	185 (122)	135 (118)	*P* < 0.001
Sweet and salty biscuits (g)	35.1 (36.2)	36.1 (35.4)	*P* = 0.49
Breakfast cereals (g)	4.5 (13.9)	3.1 (11.0)	*P* = 0.005
Whole wheat bread (g)	102 (69)	106 (91)	*P* = 0.14
White bread (g)	52.3 (66.6)	80.9 (89.6)	*P* < 0.001
Low-fat butter/margarine (g)	3.1 (3.4)	3.7 (3.9)	*P* < 0.001
Whole fat butter/margarine (g)	1.3 (2.8)	1.6 (3.2)	*P* = 0.002
Sea food salad or fish products (g)	7.6 (10.1)	8.8 (10.8)	*P* = 0.003
Meat salad or products (g)	25.9 (24.7)	38.9 (30.4)	*P* < 0.001
Cheese (g)	34.4 (28.9)	30.6 (25.1)	*P* < 0.001
Sweet spreads (g)	19.4 (19.2)	19.0 (20.1)	*P* = 0.61
Eggs (g)	8.4 (8.2)	11.5 (10.9)	*P* < 0.001
Fish/seafood (g)	14.1 (14.5)	13.8 (14.1)	*P* = 0.59
Meat substitution products (g)	1.6 (8.2)	0.9 (5.5)	*P* = 0.027
Meat/poultry/game (g)	83.4 (48.0)	89.8 (50.0)	*P* = 0.001
Whole wheat paste/whole grain rice (g)	4.0 (8.6)	4.6 (9.6)	*P* = 0.075
White pasta or rice (g)	6.9 (8.0)	7.8 (9.1)	*P* = 0.012
Deep fried potato products (g)	27.9 (25.5)	41.6 (33.9)	*P* < 0.001
Potatoes (non-deep fried, g)	162 (99)	193 (111)	*P* < 0.001
Vegetables (g)	185 (81)	167 (81)	*P* < 0.001
Sauces (g)	7.5 (7.8)	10.0 (9.3)	*P* < 0.001

Data indicate mean (standard deviation) and significance of difference as tested by Student t-test (or Welch’s t-test, if Levene’s test for homoscedasticity yielded *P* < 0.05).

**Table 3:** Associations between LTL and general dietary characteristics.

Variable	Women	Men	Total
Overall dietary score (%)	−1.05 (*P* = 0.57)	1.01 (*P* = 0.51)	0.16 (*P* = 0.89)
Diversity score (%)	−1.02 (*P* = 0.60)	2.05 (*P* = 0.27)	0.55 (*P* = 0.68)
Quality score (%)	−0.13 (*P* = 0.85)	0.56 (*P* = 0.37)	0.25 (*P* = 0.59)
Equilibrium score (%)	0.08 (*P* = 0.98)	4.09 (*P* = 0.06)	2.28 (*P* = 0.17)
Energy intake (MeJ/day)	7.68 (*P* = 0.40)	3.56 (*P* = 0.66)	5.55 (*P* = 0.36)
Fiber intake (g/day)	0.26 (*P* = 0.93)	3.72 (*P* = 0.14)	2.22 (*P* = 0.24)
DII score	26.2 (*P* = 0.32)	−30.5 (*P* = 0.26)	−1.08 (*P* = 0.95)

Data indicate beta-coefficient (*P*-value) for the continuous variable under study in a GLM adjusting for age and paternal age at birth in the female or male population, and additionally for sex in the full population, with LTL (in basepairs) as dependent variable.

### LTL and individual food items: association with deep fried potato product intake

**Figure 1:**
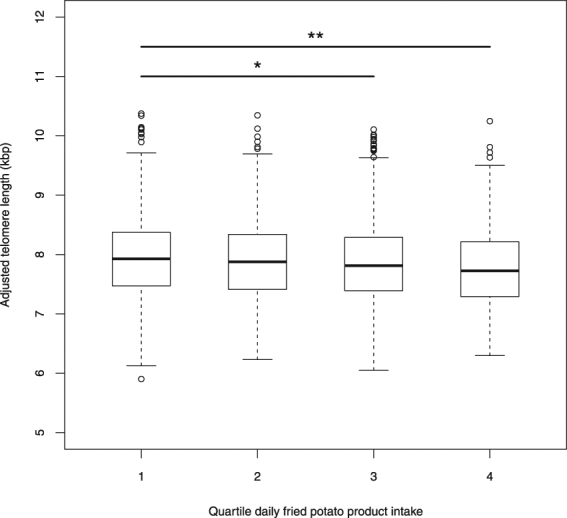
Boxplots illustrating shorter LTL (adjusted for sex, age and paternal age; in kbp, kilo base pairs) in subjects with higher daily intake of deep fried potato products (in quartiles) (*indicates *P* < 0.05; **indicates *P* < 0.005 compared to reference/first quartile). The average adjusted LTL per quartile (±standard error of the mean; in kbp) was resp. 7.94 ± 0.03 (Q1), 7.91 ± 0.03 (Q2), 7.86 ± 0.02 (Q3), 7.79 ± 0.03 (Q4). Each individual boxplot summarizes the data by depicting median, upper and lower quartile of the data (horizontal lines of the “box”) as well as the variability of data points up to 1.5 interquartile ranges outside of the upper and lower quartiles (vertical whiskers) and beyond (outliers, circles).

Subsequently, we tested the association between LTL and individual food items. Significant sex-specific associations were found between LTL and intake of several food items: for sweet and salty biscuits (positive association), and meat salad or products (positive association) in women, and for whole wheat bread (positive association), cheese (positive association) and meat/poultry/game (negative association) in men (Table 4). Though some of these results may be biologically relevant for either males or females, they may also include false positive findings, given the lack of consistency between both sexes. On the other hand, LTL was consistently inversely associated with deep fried potato product intake (i.e. French fries, deep fried potato croquettes and similar, does not include bagged chips/crisps) in both sexes (Table 4). Additional adjustment for BMI did not substantially alter the deep fried potato product – LTL association (total: *P* = 0.002, women: *P* = 0.06, men: *P* = 0.013). In the full population, LTL was significantly associated with both frequency and average portion size of deep fried potato products, which were used to calculate the average daily deep fried potato product intake (Table 4, portion size not in men). For non-deep fried potato products, there were no significant associations (*P* > 0.05 in both genders and in the full population). In line with results mentioned higher, when comparing the different quartiles of deep fried potato product intake (quartiles obtained per gender), subjects with higher consumption patterns showed modestly, but clearly significant shorter LTL (third quartile: *P* = 0.039, fourth quartile: *P* = 0.001, adjusted for age, paternal age at birth and sex) than subjects with the lowest deep fried potato product intake (first quartile) (Fig. 1). Subsequently, we set at evaluating whether oxidative stress/inflammation may mediate this association.

**Table 4:** Associations between LTL (bp) and food items.

Variable	Women	Men	Total
Sweetened or alcoholic beverages	−0.04 (*P* = 0.50)	−0.04 (*P* = 0.4)	−0.04 (*P* = 0.28)
Non-sweetened beverages	0.00 (*P* = 0.95)	−0.02 (*P* = 0.58)	−0.01 (*P* = 0.68)
Whole milk or sweetened milk products	0.24 (*P* = 0.14)	0.08 (*P* = 0.57)	0.16 (*P* = 0.14)
(Semi-)skimmed unsweetened milk products	−0.12 (*P* = 0.26)	0.07 (*P* = 0.56)	−0.04 (*P* = 0.64)
Fruits	0.14 (*P* = 0.41)	−0.02 (*P* = 0.89)	0.07 (*P* = 0.58)
Sweet and salty biscuits	1.30 (*P* = 0.020)	0.63 (*P* = 0.27)	0.97 (*P* = 0.015)
Breakfast cereals	−0.05 (*P* = 0.97)	−0.34 (*P* = 0.85)	−0.16 (*P* = 0.89)
Whole wheat bread	−0.08 (*P* = 0.78)	0.69 (*P* = 0.002)	0.41 (*P* = 0.022)
White bread	0.46 (*P* = 0.13)	−0.38 (*P* = 0.090)	−0.08 (*P* = 0.65)
Low-fat butter/margarine	0.98 (*P* = 0.87)	0.29 (*P* = 0.95)	0.42 (*P* = 0.91)
Whole fat butter/margarine	10.17 (*P* = 0.16)	4.44 (*P* = 0.48)	6.98 (*P* = 0.14)
Sea food salad or fish products	1.81 (*P* = 0.37)	−2.21 (*P* = 0.23)	−0.23 (*P* = 0.86)
Meat salad or products	2.34 (*P* = 0.004)	0.18 (*P* = 0.79)	1.10 (*P* = 0.033)
Cheese	−0.46 (*P* = 0.51)	1.90 (*P* = 0.017)	0.51 (*P* = 0.33)
Sweet spreads	1.14 (*P* = 0.28)	0.09 (*P* = 0.93)	0.58 (*P* = 0.43)
Eggs	2.46 (*P* = 0.31)	−1.32 (*P* = 0.47)	0.08 (*P* = 0.96)
Fish/seafood	−0.51 (*P* = 0.72)	1.74 (*P* = 0.22)	0.56 (*P* = 0.58)
Meat substitution products	−1.38 (*P* = 0.58)	6.73 (*P* = 0.063)	1.05 (*P* = 0.60)
Meat/poultry/game	−0.09 (*P* = 0.84)	−1.06 (*P* = 0.009)	−0.57 (*P* = 0.052)
Whole wheat paste/whole grain rice	−0.54 (*P* = 0.82)	1.15 (*P* = 0.58)	0.37 (*P* = 0.81)
White pasta or rice	−0.15 (*P* = 0.95)	−2.07 (*P* = 0.35)	−1.11 (*P* = 0.51)
Deep fried potato products	−1.58 (*P* = 0.045)	−1.51 (*P* = 0.011)	−1.51 (*P* = 0.002)
Portion size	−0.53 (*P* = 0.032)	−0.16 (*P* = 0.51)	−0.35 (*P* = 0.042)
Intake frequency	−326 (*P* = 0.115)	−392 (*P* = 0.026)	−355 (P = 0.008)
Potatoes (non-deep fried)	−0.37 (*P* = 0.073)	0.02 (*P* = 0.89)	−0.16 (*P* = 0.25)
Vegetables	−0.26 (*P* = 0.30)	0.04 (*P* = 0.87)	−0.12 (*P* = 0.51)
Sauces	0.81 (*P* = 0.76)	−0.08 (*P* = 0.97)	0.37 (*P* = 0.82)

Data indicate beta-coefficient (*P*-value) for the continuous variable under study in a GLM adjusting for age and paternal age at birth in the female or male population, and additionally for sex in the full population, with LTL as dependent variable. Beta-coefficients are expressed in bp/(g/day) or bp/(mL/day) for average daily intake variables (as listed in Table 2), and in bp/g and bp/(1/day) for average deep fried potato portion size and intake frequency respectively.

### Deep fried potato product intake and inflammation, oxidative stress and BMI

As shorter LTL is significantly associated with higher concentrations of hs-CRP, oxLDL, fibrinogen (men only), IL-6 and serum uric acid in the Asklepios Study^25^, it was evaluated whether deep fried potato product intake might be an important source of lifestyle related inflammation and oxidative stress, resulting in an indirect effect on LTL. Upon adjustment for sex, age, body mass index and current nicotine exposure in a general linear model, higher average daily deep fried potato product intake was associated with significantly (but weakly) higher concentrations of serum uric acid (P = 0.042, 0.15 mg/dl per 100 g/day) and hs-CRP (log transformed, *P* = 0.033, 0.15 ln(mg/l) per 100 g/day), but not with oxLDL (*P* = 0.51), IL-6 (*P* = 0.24), or fibrinogen (*P* = 0.43). Finally, also the association between deep fried potato product intake and BMI was evaluated. A higher average daily intake of deep fried potato products was associated with a higher BMI (*P* < 0.00001, 1.3 kg/m² per 100 g/day, age and sex-adjusted).

## Discussion

In this generally healthy, middle-aged population, only limited associations between LTL and diet could be identified. Importantly, LTL did not appear to be affected by general dietary features such as dietary quality, equilibrium and diversity, and overall dietary score. Strikingly, outcome of previously reported results regarding total energy^14^, DII^23,24^, and dietary fiber^11^ intake could not be validated. It should be noted that the food frequency questionnaire was poorly suited to assess the specific impact of other dietary components such as processed meat^12^, sugar-sweetened beverage^20^, PUFA^14,18^, vitamin D^15^ or multivitamin intake^13^.

There are several possible reasons for this general lack of significant results. A first explanation is a general lack of sensitivity of the used methodologies. Food frequency questionnaires are known to be both less accurate and precise and are subject to a variety of biases, e.g.^28^. Accuracy for assessing specific dietary indicators importantly depends on the concept and level of detail of the dietary questionnaire. Nevertheless, this methodology has been widely accepted for large-scale population studies for practical reasons. The Asklepios questionnaire is a short questionnaire (containing only 25 food items) that mainly aimed at comparing dietary intakes of the populations under study with the Food Based Dietary Guidelines, and also includes culturally specific food items such as French fries (deep fried potatoes). Because few parameters were available, this might have compromised the ability to compute e.g. reliable DII scores (45 food parameters could be included, but for 28 parameters the questionnaire lacked information, see Methods section). It is therefore plausible that a much simpler and relatively reliably reported factor such as deep fried potato intake would produce a larger observable signal. For LTL analysis, the Southern blotting methodology was used, which has the major benefit of higher reproducibility compared to the more high-throughput qPCR alternative^29^. In addition, previous results indicate sufficient sensitivity, both for LTL and dietary analysis^25–27,30^. Finally, the size of the population (~2500 subjects) and the fact that most results were clearly non-significant imply the relevance of potentially missed effects to be low in this generally healthy, middle-aged population.

The specific characteristics of the population, both regarding age and generally healthy status, provide another plausible explanation for our results. Although diet affects inflammation and oxidative stress, the impact might still be too limited in this population to be detected, especially given the limitations of the assessment tools. Similarly, although BMI and obesity have been repeatedly linked with shorter telomeres, this observation could not (yet) be made in the still relatively young Asklepios Study population^25^. Finally, it is also possible that overall dietary characteristics are not associated with LTL, a conclusion also reached by others^12^, though in a smaller and older population. This implies that, in general, the overall diet cannot be assumed to be an important driver of the shorter telomeres observed with higher levels of oxidative stress and inflammation.

Specific food items, nutrients or patterns might be more important, but it should be noted that large scale analysis of a questionnaire might easily lead to false positive results. As analogous exploratory approaches are commonplace, the multiple testing problem may provide an additional explanation for the often discordant results between studies, and why some of the reported associations could not be replicated in this study. *Vice versa*, our analyses resulted in some significant associations, typically sex-specific, that require independent validation as well (see Table 4), also because some of them appear counterintuitive. For example, the positive association between LTL and meat/poultry/game intake in men contrasts the negative association between LTL and meat salad and product consumption in women. On the other hand, it may indeed be hypothesized that the longer telomeres observed with whole wheat bread consumption in males may reflect a positive impact of dietary fiber, as previously reported (though in women^11^). Yet, when considering all sources of fiber, the association with LTL was insignificant in our study (Table 3). Nevertheless, both significant and insignificant results here reported are relevant as they may assist the interpretation of the outcome of other studies. Likewise, also the association between deep fried potato consumption and LTL was previously assessed, but not found to be significant, in a smaller qPCR based study^12^. Therefore, the significant link between LTL and deep fried potato product intake should be interpreted with caution as well, and requires additional validation. Next to the multiple testing problem, this may also simply reflect a spurious result due to yet unknown confounders. However, because LTL has been repeatedly demonstrated to be an independent – probably causal - predictor of diseases such as cancer and CVD^5–7^, successful validation may imply an impact on human health.

There are indeed several reasons why this result deserves additional consideration. The association between deep fried potato product intake and LTL was found in both men and women, but also when considering frequency and portion size (not in men) separately (Table 4). Also, deep fried potato products (which include French fries, but also deep fried potato croquettes) are very commonly consumed in Belgium, with, for example in the population under study, only 8% reporting no (or seldom) consumption of deep fried potato products. Moreover, deep fried potato product intake was a far stronger predictor of BMI than questionnaire derived daily energy intake in the population under study (data not shown). The mechanism(s) by which deep fried potato product consumption might affect LTL remains unclear. Moderate (but significant) associations were found indicating higher serum uric acid and hs-CRP concentrations with higher intake, but not for other markers of inflammation. However, it should be noted that point measurements of oxidative stress and inflammation (as used in this study) are relatively variable, e.g.^31^, and therefore only imprecise estimates of the cumulated burden of oxidative stress and inflammation. The lack of correlation with oxLDL is also consistent with the concept that circulating oxLDL is not a direct marker of oxidative stress, but rather reflects back diffusion (leakage) of oxidised LDL particles from atherosclerotic plaque into the bloodstream^32^.

It can also be hypothesized that in this population, a high level of deep fried potato product consumption can be considered as an overall proxy for the intake of deep fried foods, or even an overall unhealthy lifestyle. Indeed, next to the highly significant link with BMI, higher deep fried potato product consumption was also clearly associated (age, sex and BMI adjusted) with for example lower physical activity (frequency and intensity), lower fruit intake and higher levels of current smoking (all *P* < 0.0001), though not with vegetable intake and only borderline with alcohol intake (*P* = 0.051) (data not shown). Similarly, also in the Multi-Ethnic Study of Atherosclerosis (MESA) it was hypothesized that the significant association between LTL and processed meat (which was not available for testing in our data) may be attributed to overall lifestyle and demographic factors^12^. Finally, if these results can be validated, it should also be evaluated whether specific substances generated during the frying process, such as trans fatty acids degradation products or acrylamide, could have a direct impact on LTL.

In conclusion, LTL could not be linked with specific dietary quality parameters, and several previously reported results could not be validated in this study. Although additional validation is certainly required and the overall impact was limited, significantly longer telomeres were found in subjects with lower intake of deep fried potato products. Possible non-technical explanations for this association are the lower levels of oxidative stress and inflammation associated with low intake of deep fried potato products, the observation that high deep fried potato product consumption might be a proxy for overall unhealthy lifestyle, and direct impact of frying generated substances on LTL.

## Methods

### Study population & data collection

The Asklepios Study is a longitudinal study focusing on the interplay between ageing, cardiovascular haemodynamics and inflammation in (preclinical) cardiovascular disease, of which the baseline assessment has been completed in 2004. Based on the population lists of the twinned Belgian Erpe-Mere and Nieuwerkerken communities (provided by local authorities), subjects within the age group of 35 to 55 years (8,104 eligible) were randomly sampled and invited by tiered direct postal mailing. From the onset of the study, also partners and relatives (when living in Erpe-Mere – Nieuwerkerken) were eligible for participation. Exclusion criteria comprised clinical presence of cardiovascular disease (including clinical - i.e. symptomatic or haemodynamically significant (stenosis >50%) – atherosclerosis, previous clinical atherothrombotic events and previous or planned revascularization procedures), major concomitant illness and type 1 (and, in part, type 2) diabetes. In total, 2524 male and female, apparently healthy volunteers, were included. Subjects were extensively phenotyped, including basic clinical data, biochemical analyses of inflammation/oxidative stress markers, femoral and carotid ultrasonography, telomere length analysis and by the use of questionnaires (yielding information on physical activity and on current and past cigarette and general nicotine use). Physical activity was measured in metabolic equivalents (METs) based upon data derived from physical activity questionnaires including leisure time physical activity as well as occupational physical activity. BMI was calculated as weight (in kg) divided by the square of height (in m). The study was conducted consistent with the principles of the Declaration of Helsinki. All subjects gave written informed consent and the study was approved by the Ethical Committee of Ghent University. All methods were performed in accordance with the relevant guidelines and regulations. The datasets analysed during the current study are available from the corresponding author on reasonable request. For a full overview of the rationale, design, methods and baseline characteristics, we refer to the corresponding paper^33^.

### LTL analyses and biochemical assays

LTL was assessed using the Southern blot methodology, as described earlier^25,33^. In summary, upon isolation from whole blood (within 1 to 3 days, long term stored at −80 °C), 5 µg DNA was subjected to *Rsa*I/*Hinf*I restriction digest and field inversion gel electrophoresis. After Southern blotting, telomeric signal was visualised by hybridization with radioactively labeled probes and phosphor-imaging, and quantified using molecular weight markers as reference. Telomere length could be successfully determined in 2509 of the 2524 Asklepios Study subjects. The data from this subset constitute the basis of the study at hand. In the different analyses, information for each variable was available for at least 72% of all subjects. Validated laboratory procedures were followed to assess the concentrations of circulating biochemical markers, such as Interleukin-6 (IL-6), oxidised Low-Density Lipoprotein (oxLDL), high-sensitive C-Reactive Protein (hs-CRP), uric acid and fibrinogen, as described previously^25,33^.

### Dietary intake assessment

Asklepios participants completed a semi-quantitative food frequency questionnaire, specifically designed to assess adherence to the governmental (Flemish) food-based dietary guidelines and discussed in detail by Hoebeeck *et al*.^26^. In summary, the questionnaire included questions on the daily consumption of 25 food items during the past year, and used a semi-quantitative scale for both frequencies of consumption and portion sizes. The respondent could choose one out of six frequency categories: (almost) never, 1–3 times/month, once/week, 2–4 times/week, 5–6 times/week, once/day) and one out of three portion sizes, which were combined to estimate average daily intake. The food items included are sweetened or alcoholic beverages, non-sweetened beverages, whole milk or sweetened milk products, skimmed or semi-skimmed milk products without added sugar, fruits, sweet and salty biscuits, breakfast cereals, whole wheat bread, white bread, low-fat butter/margarine, whole fat butter/margarine, seafood salad or fish products (e.g. smoked salmon), meat salad or products (e.g. charcuterie), cheese, sweet spreads, eggs, seafood, meat substitution products, meat/poultry/game, whole wheat pasta or whole grain rice, white pasta or rice, deep fried potatoes (including French fries, deep fried potato croquettes and similar, but not bagged crisps/chips or pan fried potatoes), potatoes (non-deep fried), vegetables and sauces.

The 25 food items were aggregated into the 9 food groups included in the Flemish Food Based dietary guidelines (FBDG), considering the necessary conversion factors to make the intakes comparable with the FBDG. These 9 recommended food groups include: (1) ‘water’ (including all non-milk beverages), (2) ‘bread and cereals’, (3) ‘grains and potatoes’, (4) ‘fruit’, (5) ‘vegetables’, (6) ‘milk and milk products’, (7) ‘cheese’, (8) ‘meat, meat (substitution) products, eggs and seafood’ and (9) ‘fat’.

Based on these data, an *overall dietary index* was calculated to measure overall adherence to the FBDG, ranging from 0 to 100%. As outlined by Hoebeeck *et al*., this overall index consists of three components, being dietary diversity, quality and equilibrium^26^. The *dietary diversity score* indicates to what extent a participant eats on average at least one serving per day of the 9 food groups mentioned supra, expressed as a fraction (number of food groups from which on average at least one serving was consumed divided by 9). The *dietary quality score* shows if a person makes the optimal food choices within each of the 9 food groups. For this purpose, three categories per food group were defined in the FDBG, i.e. preference (“healthiest”), moderate and rest (“least healthy”) categories, with resp. scores 1, 0 and −1. Subsequently, a weighted score was calculated based on the corresponding portion sizes, leading to a percentage of adherence to the guidelines. The *dietary equilibrium score* indicates if an individual consumes components from the food groups in the right proportions, i.e. more/less from foods in the base/top of the food triangle. This score was calculated based on both adequacy (minimal daily recommended intake achieved or not) and moderation (maximal daily intake recommended exceeded or not), and expressed as an overall percentage of adherence to the guidelines.

Decomposition of the different food items allowed to estimate some additional general dietary characteristics, i.e. total energy and dietary fiber intake as well as the dietary inflammatory index (DII), a means to estimate the overall inflammatory potential of the diet. Nutritional (including total energy) values were assigned to each food item on the basis of weighted means of all aggregated items. The food composition data were based on the Belgian NUBEL food composition database^34^. The subject-specific total intakes of each nutrient studied, were computed by multiplying the specified frequency, portion size and nutritional value per 100 g product, subsequently summed for all food sources. This yielded total energy and dietary fiber intake estimates, but also the basis to calculate the DII, as previously described^30,35^. In summary, 17 selected inflammation-related food parameters (carbohydrate, protein, total fat, fiber, cholesterol, saturated fat, monounsaturated fat, polyunsaturated fat, *n*-6 fatty acid, thiamin, riboflavin, vitamin B12, Fe, Mg, Zn, vitamin A and vitamin C) were transformed to a z-score, converted to a centered percentile (to minimise the impact of right skewing) and subsequently multiplied by the respective food parameter specific inflammatory effect scores (derived from large scale literature review), upon which values were summed over food parameters to obtain the subject specific DII. As a result, pro-inflammatory diets are associated with higher, i.e., more positive scores, whereas more negative scores correspond to more anti-inflammatory diets.

### Statistical methods

The statistical analyses were performed using R version 3.1.0. Student’s independent t-test (function *“t.test”*), Wilcoxon rank sum-test (function “*wilcox.test*”), and the Fisher exact test (function “*fisher.test*”) were applied to test for significant differences between sexes. General Linear Models (GLM, function “*glm*”) were used to test for the significance of multiple covariates and factors in a single model. Unless explicitly mentioned otherwise, dietary variables were considered as continuous variables and average daily intake was considered as focus point for the food item analyses. In basic models evaluating association with LTL, the latter was considered as dependent variable. In these models, adjustment for age, paternal age at birth and (where relevant) sex, but not for additional variables (e.g. smoking, BMI, level of education …), was performed given that the former are important LTL determinants in the Asklepios population, whereas the latter were not significantly associated with LTL^25,27^. Only in case of significant results consistent in both sexes, portion size and frequency of consumption were analyzed separately next to average daily intake (for food item analysis); and additional adjustment was performed (i.e. for BMI). For linear models evaluating the impact on oxidative stress/inflammation related variables, adjustment for sex, age, body mass index and current nicotine exposure was performed. For hs-CRP and current nicotine use in these models, data were log-transformed (after zero-filling: for nicotine use, ln(x + 1); for hs-CRP, ln(x + 0.01), with 0.01 half of the minimum non-zero value measured). As standard regression procedures were unfit for IL-6 due to the heavily skewed and truncated distribution, IL-6 was dichotomised (cut-off: 1.5 pg/ml) and binary logistic regression was used (“*glm*” function) to assess the impact of diet on dichotomised IL-6. For the linear model with BMI as dependent variable, adjustment for age and sex was performed. Normality was not evaluated as methods were generally applicable under the central limit theorem (and skewness reducing transformations were applied where necessary). Where relevant, homoscedasticity was evaluated using the “*leveneTest*” function from the *car* package. The significance level was set at 0.05.
